# What impact have the IOC medical consensus statements made on athlete health? A survey of medical commissions from National Olympic/Paralympic Committees and International Sports Federations

**DOI:** 10.1136/bmjsem-2023-001794

**Published:** 2024-04-24

**Authors:** Lauren Victoria Fortington, Marelise Badenhorst, Wayne Derman, Carolyn Emery, Kati Pasanen, Martin Schwellnus, Evert Verhagen, Caroline F Finch

**Affiliations:** 1 Edith Cowan University, Joondalup, Western Australia, Australia; 2 Sports Performance Research Institute New Zealand (SPRINZ), Auckland University of Technology, Auckland, New Zealand; 3 IOC Research Centre South Africa, Department of Exercise, Sport and Lifestyle Medicine, Faculty of Medicine and Health Sciences, Stellenbosch University, Stellenbosch, South Africa; 4 Faculty of Kinesiology, University of Calgary, Calgary, Alberta, Canada; 5 Sport Injury Prevention Research Centre, University of Calgary Faculty of Kinesiology, Calgary, Alberta, Canada; 6 IOC Research Centre South Africa, Sport, Exercise Medicine and Lifestyle Institute (SEMLI), University of Pretoria, Faculty of Health Sciences, Pretoria, Gauteng, South Africa; 7 Amsterdam Collaboration on Health and Safety in Sports, IOC Research Centre for Prevention of Injury and Protection of Athlete Health, Department of Public and Occupational Health, Amsterdam Movement Sciences, Amsterdam UMC, Amsterdam, The Netherlands, Amsterdam, The Netherlands

**Keywords:** Sports & exercise medicine, Sporting organisation, Olympics, Knowledge translation, Consensus statement

## Abstract

**Background:**

The International Olympic Committee (IOC) Medical and Scientific Commission has supported collating and sharing evidence globally by developing sports medicine consensus statements (‘Statements’'). Publishing the Statements requires substantial resources that must be balanced by use and impact on policy and practice. This study aimed to gain a better understanding of awareness and uptake of the Statements globally through a survey of the National Olympic Committees (NOC), National Paralympic Committees (NPC) and International Federations (IF).

**Method:**

A cross-sectional survey of medical commission representatives from NOCs/NPCs/IFs. A structured questionnaire was distributed through the IOC head office, informed by prior research. Questions comprised a mix of closed and open-text responses with results presented descriptively by organisation type and total.

**Results:**

55 responses were included: 29 (52%) from NOC/NPC representatives (response rate 14%) and 26 (47%) from IF representatives (response rate 63%). All Statements had been used by at least one respondent, with the Statement addressing concussion ranked highest (used by 33/55). The main barriers to use were financial limitations (n=21), club/sport culture and behaviours (n=19) and lack of understanding from coaches/team sport personnel (n=19). Participants believed the Statements were a successful strategy for improving athlete health (n=39/51 agree or strongly agree).

**Conclusion:**

There was clear support for the continued development of sports medicine guidance, including in the format of these Statements. To ensure Statements lead to demonstrable health benefits for athletes, input from athletes, coaches and supporting staff is needed, as well as clearer identification of the purpose and audience of each topic developed.

**WHAT IS ALREADY KNOWN ON THIS TOPIC**

- There are 29 International Olympic Committee (IOC) Medical and Scientific Commission statements addressing a wide range of athlete health issues. Development of the Statements requires substantial resourcing that is ideally balanced by their use and value to athlete health globally. The impact of the IOC statements has not been evaluated.

**WHAT THIS STUDY ADDS**

- A small number of Statements have gained strong traction, whereas others have not had nearly as much influence. Participants perceived the Statements to be relevant, trustworthy, up-to-date and informative and that they provided a common understanding of evidence for decisions and medical care. Most respondents believed that the Statements are currently written for doctors and health professionals but that they should be written for everyone.

**HOW THIS STUDY MIGHT AFFECT RESEARCH, PRACTICE OR POLICY**

- There is a clear need and desire for guidance around the evidence of sports medicine. When developing Statements, the audience and purpose should be defined and a wide stakeholder group consulted, including athletes, coaches and support staff. Planning for dissemination of new Statements should also be a focus from the outset of their development. The statements are currently only published in English, and this study evaluated their use in English. Expansion of language options is recommended for the Statements and in turn, the inclusion of a more diverse user group can be included in future evaluations.

## Background

For professional athletes to achieve their peak performance, a coordinated team of coaches, managers and health professionals among others, operate behind the scenes.^1 2^ Injury or illness can be devastating; even a seemingly insignificant health problem can be the difference between success and failure.^3^ As such, the sports medicine community has focused on preventing injury and illness and understanding safe approaches to build performance.^4 5^

The International Olympic Committee (IOC) has a global focus and vision to ‘build a better world through sport’.^6^ Through its medical and scientific commission, the IOC’s goal is to ‘provide a guiding reference for all other sports organisations on matters relating to the protection of the health of athletes’.^7^ One of the ways knowledge from the IOC is shared globally is through the development and dissemination of sports medicine consensus statements (‘the Statements’). There are 29 Statements published on the IOC website (October 2023) covering a wide range of issues, from the use of platelet-rich plasma to concussion prevention and management to youth athletic development.^8^

In previous research, we reported on the academic impact^8^ and the policy/practice impact from National Olympic/Paralympic committee members of two countries.^9^ The results of those studies highlighted there was awareness of the Statements but a potential gap in their application globally. Citation impact was driven by academically well-resourced geographical regions (eg, USA, Canada, Australia, UK and Western Europe) with poor representation across much of Asia, Africa, the Middle East and Oceania.^8^ In particular, topics perceived to have the potential for ambiguous or contentious outcomes for clinical decisions were cited most (ie, concussion)^10 11^ as well as those that had supporting decision tools (eg, RED-s (Relative Energy Deficiency in Sport)).^12^ Co-publishing the Statements as peer-reviewed journal publications has also enhanced citations.^8^

The utility of the Statements on policy and best practice decisions was subsequently explored through a case study focused on two countries, finding the two settings had distinct priorities and required substantially different types of support for athletes.^9^ Australia is a high-resourced setting with a strong sports medicine industry in research, medicine and allied health.^13 14^ South Africa was chosen as a contrast to Australia in terms of health equity and resources, though notably, it also has a strong scientific influence and presence in sports medicine.^8 15^ Interviewees from Australia described an abundance of high-quality and setting/context-specific information and resources from other (non-IOC) organisations at their fingertips. At the same time, South African participants placed greater value on the IOC as a conduit for information generation and distribution. For example, South African interviewees had explained access issues, with few having university library resources, let alone those in neighbouring countries.^9^ This current study sought to establish which of those earlier case study findings were consistent across a wider range of settings and sports bodies.

To better understand awareness and uptake of the Statements globally, an international survey of representatives from the Medical Commissions of National Olympic Committees (NOCs), National Paralympic Committees (NPCs) and International Federations (IFs) of Sport was undertaken. This paper presents findings in relation to the awareness, access, application, acceptability and adoption of the Statements by these global stakeholder groups. A secondary aim was to compare findings by organisation type to determine if there were any important differences in their knowledge needs.

## Methods

### Study design

This cross-sectional study is the third component of a larger project designed to formally assess the impact of the 27 Statements published before 2019. The survey design and administration approach was based on a previous survey with the same target respondents.^5^ Participants provided their consent on the initiation of the survey questions.

### Patient and public involvement

The study addresses the issue of representation and utility of the IOC consensus statements globally with sports medicine staff as target respondents. The questionnaire was developed following a previous qualitative study in a high and low resource setting. The survey was discussed at a meeting of the IOC research centres of excellence, focusing on question wording and format. Further, five authors (WD, CE, MS, EV, CF) have been involved in the development of one or more Statements. We determined there was sufficient input to the design of the study and preferred to maximise potential participants from the NOCs/NPCs and IFs.

### Target survey respondents (participants)

Medical commission representatives of NOCs/NPCs and IFs were the survey’s target because they were the IOC’s initial intended audience for the Statements. At the time of the survey, there were 206 NOCs currently recognised by the IOC,^16^ 182 NPCs recognised by the International Paralympic Committee^17^ and 41 IFs recognised by the IOC.^18^ The IOC Medical and Scientific Department maintains an up-to-date mailing list of the positions and contact details within these committees. For this reason, the IOC facilitated the distribution of information about the survey, including the details to participate, as per our previous research.^5^ The request to participate was sent to an estimated 300 email addresses. Due to restrictions on privacy, the authors were unable to confirm any follow-up information, for example, the number of people who received and opened the email.

### Questionnaire design

A structured questionnaire was designed to be completed relatively quickly, taking approximately 15 min to complete. The questionnaire draft was developed by authors LVF, MB and CFF, informed from the citation analysis^8^ to identify the Statements of interest and interviews undertaken through the case study.^9^ The interviews provided a rich contextual understanding of some of the key issues to highlight, and the collation of interviewee discussion points was used to form the response options in the survey.

The questionnaire was designed with four sections. The first section comprised three questions. Question 1 focused on the type of organisation (NOC/NPC or IF). For NOCs, follow-up questions asked the specific country (open text) or, if unwilling to disclose, a request for the estimated size of the Olympic team (Summer, Winter, Youth) and World Bank income group (low, lower-middle, upper-middle, high). For NOCs/NPCs, there was also a question on whether there was a formal sport and exercise medicine accreditation programme. For IFs, only the respondent’s name of the federation being represented was requested (open text).

The second section (five questions) addressed broad knowledge about athlete health concerns and prior experience with the Statements. Specifically, respondents were asked to select issues they thought were relevant from a list of 37 topics derived from the interviews. Open text allowed for additional written answers. Awareness of the Statements was asked through a yes/no response. For those who were aware (responded yes), a list of the 27 Statements was presented, and respondents were asked to select all they had used. Use was defined as sharing, discussing, adapting or promoting them and informing clinical practice decisions.

The third section of the questionnaire (two questions) explored perceived barriers to the (wider) application of the Statements, from problems with access to resources to implementing their recommended actions. The final part of the questionnaire asked about beliefs in relation to the intended audience of the Statements, opportunities for improvement of their value and leadership and responsibility for athlete health (eight questions).

Questions were written in English language only. Most questions required respondents to select from various predetermined response options (see below). There was also the opportunity to provide additional free-text answers where the provided response options did not suit the respondent. Variations to the question order and wording were presented depending on the type of organisation represented (NOC/NPC or IF) and if respondents did or did not have familiarity with the Statements.

A paper version of the questionnaire was available by request from participants to the authors for those who wished to consult on responses with their colleagues. Six requests were received for a paper copy of questions; all responses were completed online. A copy of the paper version of the questionnaire is available in the online supplemental file 1.

10.1136/bmjsem-2023-001794.supp1 Supplementary data

### Survey administration

Edith Cowan University’s Survey Research Centre (SRC) in-house programming team prepared the survey for online delivery and secure data capture through November and December 2019. Pilot testing was completed for functionality with the SRC and flow and content among the author team members who were not involved in the initial development of the survey.

An invitation email and link were provided to the IOC office for distribution by email to the NOCs/NPCs and IFs. The email was addressed to the Chief Medical Officers and included the survey link, plain language information and a pdf copy of the questionnaire. A reminder email was sent after 2 weeks and again just before closing the survey at 6 weeks. The authors promoted the project and requested participation through direct contact with some organisations and discussions in professional networks.

### Data analysis

To be included in data analysis, participants must have completed the consent question and first set of questions (refer to online supplemental file 1). The questionnaire responses are presented descriptively, as frequency or proportions, and reported for total valid responses of participants, or total responses where more than one answer was allowed and split by organisation type (NOC/NPC, IF). The number of valid and missing responses is reported for each question. No statistical comparisons were conducted due to the small number of respondents. Thematic content analysis was used to code open-text responses deductively to one of the existing response options, or a new response category was established (inductively) where relevant and noted in results as such.^19^ Open-text quotes were presented (with anonymity to respond) where they enhanced the results and meaning of the quantitative findings.

## Results

### Participants

A total of 101 participants opened the questionnaire (34% of approximately 300 emails sent). 25 people did not answer any question, and 9 participants did not formally consent to participate on page 1 (n=9, 9%), so they were excluded from further analysis. Four responses were removed as they did not represent IFs or NOCs/NPCs. Eight entries from five responding NOCs/NPCs were removed as they were repeat responses from the same organisation: two provided duplicate responses and three provided triplicate (all could be confirmed as the same respondent). For two of these five respondents one entry was incomplete (the complete response was kept), and three had provided detailed more detail in open-text entries (the response with more open text was kept).

In total, 55 responses were included for analysis: 29 (52%) from NOC/NPC representatives and 26 (47%) from IF representatives.

Among NOCs/NPCs, the response rate was 14% (using the 206 NOCs as potential participants). Most NOCs/NPCs were from Europe (n=15), with eight from Eastern Europe and seven from Northern/Western Europe regions (combined). Six responses represented countries from the Americas, four from Africa, two from Asia, one from Oceania and one lower middle-income country that did not wish to disclose their location. Among IFs, the response rate was 63%. The IF representatives came from various sports that covered summer and winter sports; water and land; indoor and outdoor; judged and score-based; team and individual; and traditional and newer Olympic inclusions.

### Awareness and use

Three respondents were unaware of the Statements before taking part in the survey. Having been made aware through participation in the survey, two organisations indicated they would now consider using the Statements, and one was unsure, commenting: ‘I don’t think the medical provision is developed enough at this stage, and the NOC is small. I’ve also only been involved for a short period’.

At least one respondent had used all consensus Statements (table 1). The most used Statements were *Concussion in sport* (n=33), followed by *Sports nutrition* (n=27) and *Nutritional supplements* (n=26). The Statements that were least used were *Fasting in sports* (n=4), *Molecular basis of connective tissue and muscle injuries in sport* (n=5) and *Age determination in high-level young athletes* (n=6). No IF reported use of the two anterior cruciate ligament (ACL)-focused Statements (*Non-contact ACL in the female athlete; Prevention, diagnosis and management of paediatric ACL injuries*). *Harassment and abuse in sport* were reportedly used by more of the IFs (n=14) than NOCs/NPCs (n=8).

**Table 1:** Which of the following Statements has your organisation used

	NOC/NPC (n=29)	IF (n=26)	Combined (n=55)
Statement			
Concussion in sport	21	12	33
Sports nutrition	17	10	27
Nutritional supplements	17	9	26
Sudden cardiovascular death in sports	16	7	23
Asthma in elite athletes	13	9	22
Harassment and abuse in sport	8	14	22
Female athlete triad	12	9	21
Load in sport and risk of injury/illness	12	9	21
Periodic health evaluation of elite athletes	14	6	20
Relative energy deficiency in sport	11	7	18
Body composition, health and performance in sport	13	3	16
Mental health in elite athletes	6	9	15
Pain management in elite athletes	9	6	15
Thermoregulatory and altitude challenges in the high-level athlete	9	4	13
The use of platelet-rich plasma	8	5	13
Sex reassignment in sport	4	8	12
Youth athletic development	8	3	11
Fitness and health in young people through physical activity and sport	7	4	11
Training the elite child athlete	7	4	11
Pregnancy and the elite athlete	6	5	11
Prevention, diagnosis and management of paediatric ACL injuries	8	0	8
Prevention and management of non-communicable disease	5	3	8
Hyperandrogenism	2	6	8
Non-contact ACL injury in the female athlete	7	0	7
Age determination in high-level young athletes	4	2	6
Molecular basis of connective tissue and muscle injuries in sport	2	3	5
Fasting in sports	2	2	4
How the Statements have been used			
Mentioned in a presentation	19	13	32
Shared with medical colleagues in original format	17	12	29
Promoted among organisation for education and continuing professional development	13	12	25
Based organisation policy or formal guidelines on them	7	12	19
Used them to plan medical coverage	12	6	18
Summarised into own words/key points and shared with medical colleagues	10	8	18
Summarised into own words/key points and shared with coaches and team staff	8	10	18
Used them as training materials	11	6	17
Referred to in an organisation report or research	5	10	15
Shared with coaches and team staff in original format	9	6	15
Other	0	1	1
Barriers to using Statements			
Our financial resources are too limited	14	7	21
Club/sport culture and behaviours	9	10	19
Coaches/Sport team personnel don't understand medical issues	11	8	19
They are not practical enough for how to go about addressing the topic or issue	4	7	11
We do not have staff with the skills to understand or use them	5	4	9
They are only available in English	5	3	8
Organisational barriers from how our NOC is set up	6	1	7
Topics are not aligned with our priorities for athletes	1	5	6
They are too scientific in writing style and not easy to read or apply	2	4	6
We do not have time to apply them	3	1	4
They are not relevant to our sports or athletes	0	4	4
Don’t know how to access them	1	3	4
Not a preferred source of information	2	1	3
They are not relevant to my country or region	0	2	2
Other	2	3	5
Proposed facilitators for using Statements			
Presentations at advanced team physician course	12	11	23
Plain-language, magazine-style articles	10	13	23
Infographics	8	13	21
Publication in different languages	11	10	21
Podcasts	5	10	15
No changes required for the consensus statements as they are work for us	4	4	8
No changes are required; we will not use them in any format	0	0	0
Other	4	4	8

*Two respondents from NOCs/NPCs and two respondents from IFs did not complete these survey questions.

†Wording for those who do and do not use the Statements: Which of the following prevents you from using the Statements to a greater extent? Which of the following might prevent you from using the Statements?.

ACL, anterior cruciate ligament; IF, International Federations; NOC, National Olympic Committees; NPC, National Paralympic Committees.

The Statements were used in various ways, including presentations, education and continuing professional development (table 1). Some organisations have used them in policy or formal guidelines or to plan medical coverage. When sharing, Statements were sometimes kept in their original format and at other times summarised into their own words/key points. 11 descriptors about the Statements derived from the interviews^9^ (eg, useful, relevant, up to date) were presented to participants to indicate their agreement or disagreement. There was agreement across all response options except for ‘easy to access’, from which 17 responses were neutral (n=11) or disagreed (n=6).

Participants were asked about barriers to or greater use of the Statements in practice. The top three combined responses were financial limitations (n=21), club/sport culture and behaviours (n=19) and lack of understanding for coaches/team sport personnel (n=19). Responses from NOCs/NPCs were more often aligned to organisational barriers (eg, *barriers in financial resources* (NOC/NPC=14 vs IF=7); *organisational barriers* (NOC/NPC=6 vs IF=1)) while responses from IFs were more towards relevance of topics (eg, t*opics are not aligned to our priorities* (IF=5 vs NOCs/NPCs=1). They *are not relevant for our sports or athletes* (IF=4 vs NOC/NPC=0)). From a list of potential facilitators for increasing Statement use, popular responses were presentations at the advanced team physician course and variations in format to include plain-language, magazine-style articles and infographics.

There was also support for seeing the Statements published in different languages. Four written responses focused on making the Statements easier to access.

### Intended audience

**Figure 1:**
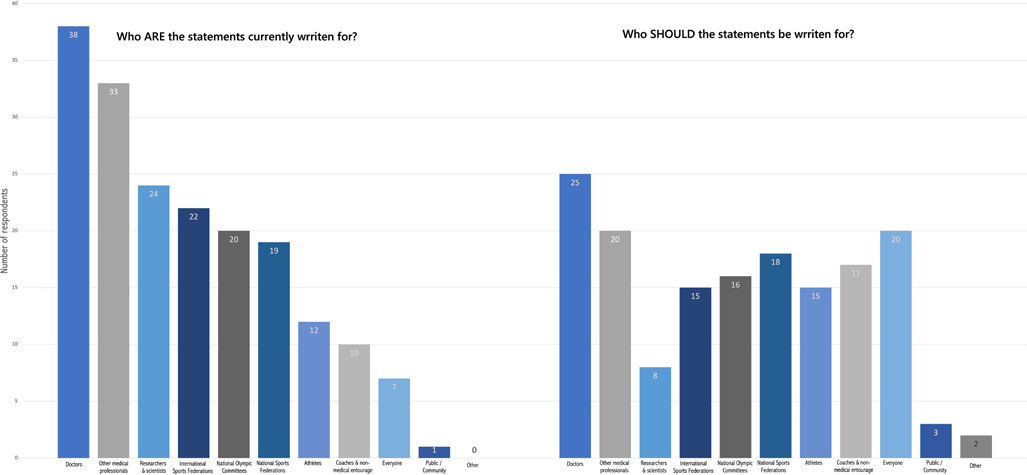
The intended audience of the Statements.

Both NOCs/NPCs and IFs provided similar responses relating to who they believed the Statements are currently written for and who the Statements should be written for. Most respondents believed that the Statements are currently written for doctors (n=38/51) and/or other medical professionals (n=33/51) (figure 1). When asked who the Statements *should* be written for, there was greater variation in responses, with 20 respondents selecting ‘everyone’.

### Improving athlete health

Participants largely believed the Statements serve the purpose of improving athlete health (n=39/51 agree or strongly agree) (table 2). Almost half (24/51) of the participants thought the IOC Statements were, or could be, useful to improve the health of athletes, the same number were unsure (n=24), and three disagreed (n=3). Overall, both NOC/NPC and IF respondents considered the Statements to provide useful supporting evidence and a common understanding on which to base discussion, decisions, guidelines and medical care. Two examples of responses were:

- *It’s easier to introduce and improve our medical guidelines with the support of IOC medical consensus statements*.
- *The consensus guidelines are a critical source of current best practices, especially for concussion: because so little is known and because it is changing SO rapidly*.

**Table 2:** Improvement in athlete health from the Statements

Statement	NOC/NPC (n=27*)	IF (n=24*)	Combined (n=51)
How well do you think the Statements serve the purpose of improving athlete health?			
Not at all/a little	3	3	6
Somewhat (neutral)	1	3	4
Quite well	17	11	28
Very much	5	6	11
Do not know	1	1	2
Have the Statements improved athlete health?			
Yes	12	12	24
No	1	2	3
Do not know	14	10	24

*There were two respondents from NOCs/NPCs and two respondents from IFs who did not complete these questions of the survey.

IF, International Federations; NOC, National Olympic Committees; NPC, National Paralympic Committees.

## Discussion

This survey confirms previous findings that some Statements have gained strong traction, whereas others have not had nearly as much influence. Most notably, *the concussion in sport statement* was found to have a strong citation impact, had good awareness among South African and Australian NOC/NPC representatives and was the most used Statement identified by NOCs/NPCs and IFs globally. It was not clear from this survey why some Statements were preferred over others. However, our previous case study suggested a leaning toward topics with the potential for controversial direction debate or uncertainty in clinical decisions.^9^ Two of the Statements reportedly least used addressed ACL injury,^20 21^ an injury with substantial clinical guidance available and, arguably, relatively clear prevention, treatment and rehabilitation pathways.^22 23^

Participants were generally positive toward a range of Statement descriptors, perceiving them to be *relevant*, *trustworthy*, *up-to-date* and *informative*, among other features. The weakest result was seen for *ease of access,* with 17 responses being neutral or negative. Improving access was also notable in the written responses to potential facilitators of use. Challenges with accessing the Statements confirm our findings from the case study.^9^ While we cannot disclose which specific organisations selected these responses, there was no discernible pattern in the location or sport with European, African and South American countries represented, as well as sports from both Summer and Winter events and individual and team pursuits. Thus, a broad strategy to support dissemination and accessibility to all IFs and NOCs/NPCs is needed.

Dissemination is the ‘purposive distribution of a guideline to a specific audience to enhance awareness, attitudes, and knowledge of a guideline’.^24^ One successful dissemination strategy that has seen increased readership for the documents was to co-publish them as open-access peer-reviewed journal publications.^8^ However, this strategy targets those more academically inclined and confident to read a scientific-style journal. Beyond the peer-reviewed publications, it could be argued that the *purposive* element has been largely missing in disseminating the Statements (with more of a passive ‘diffusion’ process wherein the information is simply provided with no active planning.) A good dissemination plan will consider a range of strategies that address the audience, the message, the format and who will deliver this. Potentially, a lack of clarity on these features underlies some of the difficulty in dissemination and, in turn, access and use of the Statements.

Survey respondents indicated the Statements are written for doctors and allied health practitioners; however, they believe they should be written for everyone, including team staff, management and athletes. Establishing clarity with the audience aligns closely with a clear purpose or aim statement. Identified through our earlier work, interviewees asked, ‘What is the intention of the statements? Who are they for?’^9^ Different audiences—and different document purposes—will demand different strategies for dissemination. It is necessary to be clear on these directions before considering the best ways to support expanding reach and use.^25^ The intended audience, whether athletes or coaches, for example, should also be involved in their design and development.

In 2023, the WHO reported on a workshop focused on principles to improve the usability and impact of WHO guidelines.^26^ The seven design principles featured (eg, principle 2—design for access and accessibility) are potentially a good starting place for the Statements targeting the same intended global audience to ‘provide recommendations on public health policy or for health interventions’ and ‘to ensure the usability and impact of these products in countries’.^26^ In order to meet the needs of ‘everyone’ as indicated by respondents, an initial solution for the IOC Statements, could be the co-production of plain language summaries targeted at athletes or a community audience, designed with their input.

The development of the Statements has changed since 2019 and there has been a shift to include authors from wider geographical representation and transparency in their development, for example, seeking nominations to contribute to development^27^ using an online survey for input from a global audience^28^ and transparent reporting of methods.^29^ Follow-up will be important to evaluate how these changes influence the future use and acceptability of the Statements, and ultimately, if they result in their intended improvement to athlete health.

This descriptive, cross-sectional study has several limitations to consider. A survey design was chosen as it enabled a cost-effective method to include sporting and medical commissions from across the world. Two preceding studies informed the questions but were not formally tested for reliability. A pdf version of the questionnaire was provided so respondents could consider their responses and discuss them with teams before submitting them. Five NOCs/NPCs provided repeat responses (same respondent); two were incomplete (the complete response was kept), and three had open text in detail (this response with more open text was kept). While small differences for these five repeat respondents were observed in the selected item responses, these were not large enough to make important changes to results or conclusions.

The included organisations were from a variety of geographical regions and sports but overall, there was a smaller than anticipated number of survey respondents. We do not have information on why emails were not opened or responded to by potential participants. While lower than desired, 55 medical officers from high-performance sport in a study of this nature is valuable and comparable to previous research.^5^ There was low inclusion from non-English speaking geographical areas and there remains a need to identify the sports medicine resources that are currently used and most impactful in these regions. We hope this evaluation will inform a broader analysis of resources (beyond the IOC statements) that are useful to a range of organisations, extending from the NOC/NPCs and IFs. While unique insights will differ, the take-home messages of needing clarity of audience and purpose to support purposive dissemination are unlikely to be impacted by the inclusion of additional responses.

The survey was provided only in English, on the basis that international sports medicine practitioners will likely be familiar though the language barrier is still important to consider. Some open-text responses were provided in French (which the authors could understand sufficiently to translate) suggesting the questions may not have been straightforward for all representatives and understanding some concepts is difficult. It is possible that respondents’ prior interactions with the IOC influenced their choice to respond (or not) and how they interpreted the questions. We do not know how many Paralympic committees were involved in responses, though we believe them to be under-represented. We also do not have confirmation that the responses were provided or informed by the medical officers, despite them being our target respondent. Ideally, being able to link directly with potential participants could help with representation from all groups, however, this was not possible with our preference to maintain anonymity of respondents. This research considered only the IOC-supported Statements, and readers may not separate the use of consensus guidelines from the organisation that funds or supports their development. Potentially, responses about Statements may be mixed up with Statements from other organisations.

## Conclusion

There appears to be a clear need and desire for sports medicine guidance documents like the Statements considered in this work. To ensure they lead to demonstrable health benefits for athletes, however, any ongoing development of such Statements and strategies for their implementation should engage a wide audience. The audience could include one or more of a range of interested groups stakeholders such as athletes, coaches, support staff, parents, administrators or referees.

We have previously documented the limited authorship representation in the published Statements.^8^ For the increased global reach of this important health guidance, it is strongly recommended that the IOC consider more opportunities for contributions to the Statements from relevant experts outside Western Europe, the USA, Australia, Canada and Scandinavia.

For those wishing to develop sports medicine guidelines in the future, the following recommendations are made with respect to their preparation:

- Identify their purpose/s up-front and clearly state the purpose of each statement.
- Consider the target audience for each tailored output, and state this clearly. Some high-resource countries may not require information on certain topics in contrast to low-resource settings, which may strongly desire these topics.
- Plan, define and prepare associated documents in multiple formats to support reach to different audiences. Exploration of the requirements of the low/high resource settings in knowledge availability more generally should inform decisions about dissemination. Tailored and targeted information for coaches and athletes is particularly important and should include infographics, videos and other non-written formats.
- Consider creating a uniform identity across guidelines and their linked resources. Present consistent terminology and branding on them to enable recognition and ensure confidence in the quality of the information being presented.

Importantly, planning for dissemination of guidelines needs to be a focus from the outset. Incorporating this planning through the stages above will ensure that the developed guidelines will have maximal benefit for their intended users.

## Data Availability

All data relevant to the study are included in the article or uploaded as supplementary information.

## References

[1] Smyth EA , Donaldson A , Drew MK , et al . What contributes to athlete performance health? A concept mapping approach. Int J Environ Res Public Health 2022;20:300. 10.3390/ijerph20010300 36612621 PMC9819660

[2] Burns L , Weissensteiner JR , Cohen M . Lifestyles and Mindsets of Olympic, Paralympic and world champions: is an integrated approach the key to elite performance Br J Sports Med 2019;53:818–24. 10.1136/bjsports-2018-099217 30352862

[3] Raysmith BP , Drew MK . Performance success or failure is influenced by weeks lost to injury and illness in elite Australian track and field athletes: a 5-year prospective study. J Sci Med Sport 2016;19:778–83. 10.1016/j.jsams.2015.12.515 26839047

[4] Dijkstra HP , Pollock N , Chakraverty R , et al . Managing the health of the elite athlete: a new integrated performance health management and coaching model. Br J Sports Med 2014;48:523–31. 10.1136/bjsports-2013-093222 24620040 PMC3963533

[5] Finch CF , Talpey S , Bradshaw A , et al . Research priorities of international sporting federations and the IOC research centres. BMJ Open Sport Exerc Med 2016;2:e000168. 10.1136/bmjsem-2016-000168 PMC5125424 27900197

[6] IOC - International Olympic Committee, Olympics.com . International Olympic Committee. 2022. Available: https://olympics.com/ioc/overview [Accessed 16 Jan 2023].

[7] Medical and Scientific Commission . International Olympic committee. 2022. Available: https://olympics.com/ioc/medical-and-scientific-commission [Accessed 6 Sep 2022].

[8] Fortington LV , Handcock RN , Derman W , et al . The citation impact and reach of the IOC sport and exercise medicine consensus statements. BMJ Open Sport Exerc Med 2023;9:e001460. 10.1136/bmjsem-2022-001460 PMC9896205 36741789

[9] Fortington LV , Badenhorst M , Bolling C , et al . Are we levelling the playing field? A qualitative case study of the awareness, uptake and relevance of the IOC consensus statements in two countries. Br J Sports Med 2023;57:1371–81. 10.1136/bjsports-2022-105984 36725283 PMC10646857

[10] International Olympic Committee . IOC - medical and scientific consensus statements - concussion in sport. 2023. Available: https://olympics.com/ioc/documents/athletes/medical-and-scientific-consensus-statements [Accessed 7 Jul 2023].

[11] McCrory P , Meeuwisse W , Dvořák J , et al . Consensus statement on concussion in sport-the 5th international conference on concussion in sport held in Berlin, October 2016. Br J Sports Med 2017;51:838–47. 10.1136/bjsports-2017-097699 28446457

[12] Mountjoy M , Sundgot-Borgen J , Burke L , et al . The IOC consensus statement: beyond the female athlete Triad—relative energy deficiency in sport (RED-S). Br J Sports Med 2014;48:491–7. 10.1136/bjsports-2014-093502 24620037

[13] Sports Medicine Australia . Australia’s leading multi-disciplinary sports medicine body. Available: https://sma.org.au/ [Accessed 7 Jul 2023].

[14] Australasian College of Sport and Exercise Physicians . Sport and exercise medicine (SEM). Available: https://www.acsep.org.au/page/about/SEM [Accessed 7 Jul 2023].

[15] The South African Sports Medicine Association (SASMA) . The South African sports medicine Association (SASMA). Available: https://www.sasma.org.za/ [Accessed 7 Jul 2023].

[16] National Olympic Committees (NOC) - Olympic Movement . International Olympic committee. 2023. Available: https://olympics.com/ioc/national-olympic-committees [Accessed 9 Oct 2023].

[17] National Paralympic committees. Available: https://www.paralympic.org/teams-npc [Accessed 9 Oct 2023].

[18] International Olympic Committee . Recognised federations. 2023. Available: https://olympics.com/ioc/recognised-international-federations [Accessed 9 Oct 2023].

[19] Rouder J , Saucier O , Kinder R , et al . What to do with all those open-ended responses? Data visualization techniques for survey researchers. Surv Pract 2021;14:1–9. 10.29115/SP-2021-0008

[20] Ardern CL , Ekås G , Grindem H , et al . International Olympic committee consensus statement on prevention, diagnosis, and management of pediatric anterior Cruciate ligament injuries. Orthop J Sports Med 2018;6:2325967118759953. 10.1177/2325967118759953 29594177 PMC5865521

[21] Renstrom P , Ljungqvist A , Arendt E , et al . Non-contact ACL injuries in female athletes: an international Olympic committee current concepts statement. Br J Sports Med 2008;42:394–412. 10.1136/bjsm.2008.048934 18539658 PMC3920910

[22] Arundale AJH , Silvers-Granelli HJ , Myklebust G . ACL injury prevention: where have we come from and where are we going J Orthop Res 2022;40:43–54. 10.1002/jor.25058 33913532

[23] Gokeler A , Grassi A , Hoogeslag R , et al . Return to sports after ACL injury 5 years from now: 10 things we must do. J Exp Orthop 2022;9:73. 10.1186/s40634-022-00514-7 35907095 PMC9339063

[24] Rabin BA , Brownson RC , Haire-Joshu D , et al . A glossary for dissemination and implementation research in health. J Public Health Manag Pract 2008;14:117–23. 10.1097/01.PHH.0000311888.06252.bb 18287916

[25] McCormack L , Sheridan S , Lewis M , et al . Communication and dissemination strategies to facilitate the use of health-related evidence. Evid Rep Technol Assess (Full Rep) 2013;1–520. 10.23970/ahrqepcerta213 PMC4781094 24423078

[26] World Health Organisation . Improving the usability and impact of WHO guidelines: report of a WHO workshop. Available: https://www.who.int/publications-detail-redirect/9789240057029 [Accessed 9 Oct 2023].

[27] Schwellnus M , Adami PE , Bougault V , et al . International Olympic Committee (IOC) consensus statement on acute respiratory illness in athletes part 1: acute respiratory infections. Br J Sports Med 2022;56:1066–88. 10.1136/bjsports-2022-105759 35863871

[28] Bahr R , Clarsen B , Derman W , et al . International Olympic committee consensus statement: methods for recording and reporting of epidemiological data on injury and illness in sport 2020 (including STROBE extension for sport injury and illness surveillance (STROBE-SIIS)). Br J Sports Med 2020;54:372–89. 10.1136/bjsports-2019-101969 32071062 PMC7146946

[29] Patricios JS , Schneider KJ , Dvorak J , et al . Consensus statement on concussion in sport: the 6th international conference on concussion in sport–Amsterdam, October 2022. Br J Sports Med 2023;57:695–711. 10.1136/bjsports-2023-106898 37316210

