# Supplementary material for: What impact have the IOC medical consensus statements made on athlete health? A survey of medical commissions from National Olympic/Paralympic Committees and International Sports Federations
Source: BMJ Open Sport Exerc Med. 2024 Apr 24;10(2):e001794. doi: 10.1136/bmjsem-2023-001794 (PMC11043694; doi:10.1136/bmjsem-2023-001794)
Supplement: Supplementary data [file bmjsem-2023-001794supp001.pdf]

Thank you for participating in this survey.

The IOC Medical and Scientific Commission provides a reference point for other sporting organisations on matters relating to the protection of athlete health. In undertaking this role, the Commission has outlined responsibilities, which include delivering evidence-based education to athletes and their entourage. One of the strategic actions for the Commission to achieve this objective is **to maximise the relevance and the dissemination of the consensus statements and publications.**

The number of consensus statements has grown, however the dissemination (where the documents have been used, by whom, and how) and the implementation of these documents (how the documents are used and what the outcomes of them are) has not been evaluated. This project is part of an evaluation of these statements.

The questions address how your organisation has engaged with the IOC consensus statements and publications. Your responses will help us understand how the resources are used and how athlete health can best be protected.

Fortington LV, *et al. BMJ Open Sp Ex Med* 2024; 10:e001794. doi: 10.1136/bmjsem-2023-001794

SECTION 1

Q1

Are you reporting this survey as a representative of a National Olympic Committee or an International Sport Federation?

☐ National Olympic Committee.

☐ International Federation. *Please cease completion and request the IF version*

Q2

We would like to understand a little more about the country your organisation represents particularly the size and resources that are available. Could you please let us know the name of your country?

☐ Country____

Q3 **In your country, is there a formal accreditation process to become a sport and exercise physician? *Select one option***

☐ There is a formal accreditation process (exams, training) and / or governing body specifically for sport and exercise physicians

☐ There is a formal accreditation process for medical training but not specifically for sport and exercise physicians

☐ No

☐ Unsure

SECTION 2 -

Q1

Please select from the following, any of the issues or concerns with athlete health that your organisation view as important or potentially important in the future:

Choose as many options as relevant

Mark with X	Possible topics	Mark with X	
	Academy/developing athletes		Non-sporting career development (jobs and study)
	Youth mental health		Athlete non-disclosure of injury/illness (desire to compete at all costs)
	Athlete lifestyle (managing daily stresses)		Supplement use
	Health provider (medical staff) health and wellbeing		Access to athlete health records
	Athlete wellbeing (personal relationships)		Conflicts with coaches on medical decisions
	Alternative medicines		Mental health
	Long term joint health and osteoarthritis		Anti-doping
	Advancements in genetic sciences		Immediate care of athletes after an event
	Knee ligament injury		Management of athletes between major events
	Serious injury in extreme (high-risk) sports		Concussion
	Basic hygiene		Child protection
	Dermatology - skin health		Behaviours for making weight
	Medical insurance access		General practice issues – everyday health care
	Equality of health care for all athletes		Early specialisation of athletes
	Gut health		Terror incidents
	Inclusion of diverse gender athletes		Management of athletes during events
	Exertional heat illnesses		Disordered eating
	Screening and care of athletes immediately pre-event		Athlete-coach relationships
	Traditional medicines (cultural / indigenous beliefs and		
Other. *Please list.*			

Fortington LV, *et al. BMJ Open Sp Ex Med* 2024; 10:e001794. doi: 10.1136/bmjsem-2023-001794

SECTION 2
Q2

Before receiving this survey, were you aware the IOC has a series of published Medical Consensus Statements?

☐ **YES.** *Which consensus statements listed below has your organisation used? Use can include tasks such as sharing, discussing, adapting or promoting them.*

☐ **NO / NOT SURE.** *The IOC has supported the development of the following Consensus Statements addressing athlete health. Please indicate which topics listed below you think are of interest for your organisation.*

Q3

Please choose as many responses as relevant

Mark with X	IOC Medical Consensus Statement Topic
	Sex reassignment in sport
	Sudden cardiovascular death in sports
	Female athlete triad
	Training the elite child athlete
	Harassment and abuse in sport
	Molecular basis of connective tissue and muscle injuries in sport
	Asthma in elite athletes
	Non-contact ACL injury in the female athlete
	Fasting in sports
	Periodic health evaluation of elite athletes
	Sports nutrition
	The use of platelet-rich plasma
	Fitness and health in young people through physical activity and sport
	Body composition, health and performance in sport
	Concussion in sport
	Prevention and management of non-communicable disease
	Youth athletic development
	Hyperandrogenism
	Pregnancy and the elite athlete

	Mental health in elite athletes
	Relative energy deficiency in sport (RED-S)
	Load in sport and risk of injury/illness
	Pain management in elite athletes
	Nutritional supplements
	Prevention, diagnosis & management of paediatric ACL injuries
	Thermoregulatory and altitude challenges in the high-level athlete
	Age determination in high-level young athletes

Q4

Are these statements something you might now consider using in your organisation?

- ☐ Already using
- ☐ Yes
- ☐ No
- ☐ Not sure

Q5

If yes, please indicate why you WOULD consider using these statements
If no or not sure, Please indicate why you are not sure if you would consider using these statements. ***Select as many responses as relevant***

- ☐ Trustworthiness
- ☐ Accuracy
- ☐ Informative
- ☐ Usefulness
- ☐ Relevance
- ☐ Accessibility
- ☐ Prefer other resources – please specify:___________________________________
- ☐ Other reason – please specify:____

Q4 alternate

In which of the following ways has your organisation used the consensus statements? *Please select as many as relevant.*

- ☐ shared with medical colleagues in their original format
- ☐ shared with coaches/team staff in their original format
- ☐ summarised into own words/key points and shared with medical colleagues
- ☐ summarised into own words/key points and shared with coaches/team staff
- ☐ promoted in organisation for education / continuing professional development
- ☐ referred to in organisation reports or research
- ☐ mentioned in a presentation
- ☐ used them to plan medical coverage
- ☐ used them as training materials
- ☐ based organisational policy or formal guidelines on them
- ☐ have not used them
- ☐ other – please describe ________________________________

Q5 alternate

How strongly do you believe the IOC Consensus Statements are:

**Trustworthy**	not at all	a little	somewhat	quite	very much	don't know
**Accurate**	not at all	a little	somewhat	quite	very much	don't know
**Informative**	not at all	a little	somewhat	quite	very much	don't know
**Useful**	not at all	a little	somewhat	quite	very much	don't know
**Relevant**	not at all	a little	somewhat	quite	very much	don't know
**Easy to access**	not at all	a little	somewhat	quite	very much	don't know
**Easy to understand**	not at all	a little	somewhat	quite	very much	don't know
**Covering current issues/concepts**	not at all	a little	somewhat	quite	very much	don't know
**Consistent**	not at all	a little	somewhat	quite	very much	don't know
**Suited to your context**	not at all	a little	somewhat	quite	very much	don't know
**Up to date**	not at all	a little	somewhat	quite	very much	don't know

Fortington LV, *et al. BMJ Open Sp Ex Med* 2024; 10:e001794. doi: 10.1136/bmjsem-2023-001794

SECTION 3
Q1

Which of the following prevents you from using the resources to a greater extent than currently? Or, which of the following *might* prevent you from using the IOC consensus statements? *Please select as many as relevant*

- ☐ Topics are not aligned to our priorities for athletes
- ☐ Organisational barriers from how our National Olympic Committee is set up
- ☐ Our financial resources are too limited
- ☐ Club/sport culture and behaviours
- ☐ Coaches/Sport team personnel don't understand medical issues
- ☐ We do not have time to apply them
- ☐ They are not relevant for our sports or our athletes
- ☐ They are only available in English
- ☐ They are too scientific in writing style and not easy to read or apply
- ☐ They are not practical enough for how to go about addressing the topic or issue
- ☐ We do not have staff with the skills to understand or use them
- ☐ Don't know how to access them
- ☐ Not a preferred source of information
- ☐ They are not relevant for my country or region
- ☐ Other – please describe below -

____
____
____

Q2

Have the IOC medical consensus statements improved the health of athletes that are part of your organisation? Or, do you think the IOC medical consensus statements could be useful to improve the health of athletes in your organisations care?

- ☐ Yes – please specify why:
- ☐ No – please specify why:
- ☐ Don't know

SECTION 4

Q1

How well do you think the statements serve the purpose of improving athlete health?

- ☐ not at all
- ☐ a little
- ☐ somewhat
- ☐ quite
- ☐ very much
- ☐ don't know

Q2

Who do you think the statements are written and designed for?
Please select as many as relevant

- ☐ Everyone
- ☐ Doctors
- ☐ Athletes
- ☐ Coaches and non-medical athlete entourage (e.g. team manager)
- ☐ Other medical staff and professional networks (e.g. physical therapist, nutrition, strength & conditioning)
- ☐ International Sports Federations
- ☐ National Sports Federations
- ☐ National Olympic Committees
- ☐ Public / community members
- ☐ Researchers and scientists
- ☐ Other - please specify:

____
____
____
____

SECTION 4
Q3

Who do you think the statements SHOULD be written and designed for?
Please select as many as relevant

- ☐ Everyone
- ☐ Doctors
- ☐ Athletes
- ☐ Coaches and non-medical athlete entourage (e.g. team manager)
- ☐ Other medical staff and professional networks (e.g. physical therapist, nutrition, strength & conditioning)
- ☐ International Sports Federations
- ☐ National Sports Federations
- ☐ National Olympic Committees
- ☐ Public / community members
- ☐ Researchers and scientists
- ☐ Other - please specify: ____

Q4

Which of the following would best help your organisation in using the consensus statements to manage athlete health?

- ☐ No changes required, the consensus statements as they are work for us
- ☐ No changes, we will not use them in any format

Or, please select as many as relevant from below

- ☐ Presentations at advanced team physician courses
- ☐ Podcasts
- ☐ Infographics
- ☐ Plain-language, magazine-style articles
- ☐ Publication in different languages
- ☐ Other - please specify:

____

____

Q5a

Please indicate which of the following groups should have the main leadership role in IDENTIFYING PRIORITIES for athlete health. *Please do this by writing the numbers 1 – 9 (1 being main leadership role, 9 being lowest) alongside each response choice.*

- ☐ Doctor and professional networks
- ☐ Athletes
- ☐ Coaches and non-medical athlete entourage (e.g. team manager)
- ☐ Other medical staff and professional networks (e.g. physical therapist)
- ☐ International Sports Federations
- ☐ National Sports Federations
- ☐ International Olympic Committee
- ☐ National Olympic Committee
- ☐ Researchers and scientists

Q5b

Aside from the 9 groups just mentioned, is there ANOTHER group that you believe should have a leadership role in IDENTIFYING PRIORITIES for athlete health?

- ☐ Yes - please specify:________________________________
- ☐ No

Q5c

Which ranking should this group have - where would they sit in the above list of leadership in IDENTIFYING PRIORITIES for athlete health, compared to the other 9 groups. *Please choose one ranking (1 to 10)*

☐

1 – highest leadership role through **10** – lowest leadership role

Q6a

Please indicate which of the following groups should have the main leadership role in PROVIDING KNOWLEDGE AND INFORMATION for athlete health. *Please do this by writing the numbers 1 – 9 (1 being main leadership role, 9 being lowest) alongside each response choice*

- ☐ Doctor and professional networks
- ☐ Athletes
- ☐ Coaches and non-medical athlete entourage (e.g. team manager)
- ☐ Other medical staff and professional networks (e.g. physical therapist)
- ☐ International Sports Federations
- ☐ National Sports Federations
- ☐ International Olympic Committee
- ☐ National Olympic Committee
- ☐ Researchers and scientists

Q6b

Aside from the 9 groups just mentioned, is there ANOTHER group that you believe should have a leadership role in PROVIDING KNOWLEDGE AND INFORMATION for athlete health?

- ☐ Yes - please specify:________________________________
- ☐ No

Q6c

Which ranking should this group have - where would they sit in the above list of leadership in IDENTIFYING PRIORITIES for athlete health, compared to the other 9 groups. *Please choose one ranking (1 to 10)*

☐

1 – highest leadership role through **10** – lowest leadership role

Q7

Are there other topics on athlete health that should be addressed in a future IOC Consensus Statement?

____
____
____
____
____
____

Q8

Do you have any other feedback on the consensus statements or athlete health that you wish to share?

____
____
____
____
____
____

Thank you.

Your responses to this study are most appreciated. The results will be reviewed and drawn together with other research findings to inform the International Olympic Committee Medical and Scientific Commission on the awareness and use of the Consensus Statements.

If you wish to receive a plain language summary of the results, please provide your email below. Your email address will not be linked to your survey responses.

Email :______________________________@__________________________________

----end survey ---

Please scan this document and email your responses to ACRISP@ECU.EDU.AU

Fortington LV, *et al. BMJ Open Sp Ex Med* 2024; 10:e001794. doi: 10.1136/bmjsem-2023-001794
